# Intraprostatic injection of botulinum toxin type- A relieves bladder outlet obstruction in human and induces prostate apoptosis in dogs

**DOI:** 10.1186/1471-2490-6-12

**Published:** 2006-04-18

**Authors:** Yao-Chi Chuang, Chieh-Hsien Tu, Chao-Cheng Huang, Hsin-Ju Lin, Po-Hui Chiang, Naoki Yoshimura, Michael B Chancellor

**Affiliations:** 1 Department of Urology, Chang Gung Memorial Hospital Kaohsiung Medical Center, Chang Gung University College of Medicine, Kaohsiung, Taiwan; 2 Department of Veterinary Medicine, National Pingtung University of Science and Technology, Taiwan; 3 Department of Pathology, Chang Gung Memorial Hospital Kaohsiung Medical Center, Chang Gung University College of Medicine, Kaohsiung, Taiwan; 4 Department of Urology, University of Pittsburgh School of Medicine, Pittsburgh, Pennsylvania, USA

## Abstract

**Background:**

With the increasing interest with botulinum toxin – A (BTX-A) application in the lower urinary tract, we investigated the BTX-A effects on the canine prostate and also in men with bladder outlet obstruction (BOO) due to benign prostatic hyperplasia (BPH).

**Methods:**

Transperineal injection into the prostate using transrectal ultrasound (TRUS) was performed throughout the study. Saline with or without 100 U of BTX-A was injected into mongrel dogs prostate. One or 3 months later, the prostate was harvested for morphologic and apoptotic study. In addition, eight BPH patients refractory to α-blockers were treated with ultrasound guided intraprostatic injection of 200 U of BTX-A.

**Results:**

In the BTX-A treated dogs, atrophy and diffuse apoptosis was observed with H&E stain and TUNEL stain at 1 and 3 months. Clinically, the mean prostate volume, symptom score, and quality of life index were significantly reduced by 18.8%, 73.1%, and 61.5% respectively. Maximal flow rate significantly increased by 72.0%.

**Conclusion:**

Intraprostatic BTX-A injection induces prostate apotosis in dogs and relieves BOO in humans. It is therefore a promising alternative treatment for refractory BOO due to BPH.

## Background

Benign prostatic hyperplasia (BPH) is a common feature of the aging male and is regarded as a major cause of bladder outlet obstruction (BOO), which results in obstructive and irritative voiding symptoms. The bothersome urinary symptoms decrease the quality of life of these patients [1]. Transurethral resection of prostate (TURP) has been considered as a gold standard for the treatment of symptomatic BPH. However, there have been concerns about the safety of TURP, with long-term morbidity including retrograde ejaculation, bladder neck contracture, and impotence [2-4]. Thus, there has been much interest in the development of alternative treatments. However, one feature of any of the minimally invasive techniques in clinical use to date is that they are permanent. Some men are simply not ready or willing to undergo an irreversible procedure in order to treat their symptomatic BPH.

Botulinum toxin A (BTX-A) has been shown to block the release of neurotransmitters such as acetylcholine at the neuromuscular junction as well as in autonomic neurons [5-7]. BTX-A has been successfully used to treat various conditions including blepharospasm, strabismus, focal dystonias, and achalasia [7]. Injection of BTX-A into the urethra or bladder is an effective treatment for various types of lower urinary tract dysfunction for 6 months or longer [7-9]. Furthermore, previous studies in the rats have shown that intraurethral injection of BTX-A has significant inhibitory effects on urethral norepinephrine release [10] and intraprostatic injection of BTX-A induces selective denervation and subsequent atrophy of the glands [11]. Recent clinical studies have shown promising results after injection of BTX-A into lateral lobes of prostate for relieving voiding dysfunction due to BPH [12-14]. However, the histological change of prostatic tissue and mechanisms after BTX-A injection was not clear. The dog is one of a few animals that can develop BPH spontaneously and is frequently used as an animal model for human prostatic hyperplasia [15,16]. Therefore, the present study investigated morphologic and apoptotic change in the canine prostate after BTX-A injection. We also reported 8 cases of symptomatic BPH patients treated with BTX-A.

## Methods

### Animal study

**Figure 1:**
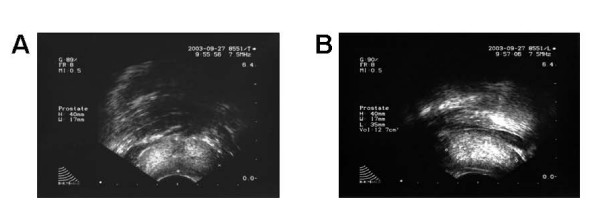
Diffusion of hyperechoic BTX-A over the canine prostate immediate after BTX-A injection. A. Transverse view. B. Longitudinal view.

The animal study was conducted at veterinary hospital, National Pingtung University, with the approval of the institutional review board. Eight mature male mongrel dogs, weighing 13 to 17 kg., were used in this study. All animals were put on the lithotomy position and received perioperative antibiotics and intravenous sedation with pentobarbital. One hundred units of botulinum toxin A (BTX-A, Botox^®^, Allergan, Irvine CA) were dissolved in 4 ml of 0.9% saline solution. Each lobe of the prostate received transperineal injection of 2 ml of BTX-A with one-needle pass under TRUS guidance (Fig. 1) [16,17]. The control animals received the same procedures with injection of normal saline.

One or three months after BTX-A or normal saline injection (N = 2, in each group), the prostate was harvested for histological examination. The prostatic tissues were embedded in paraffin blocks after formalin fixation. Consecutive sections were taken for morphologic examination with hematoxylin and eosin stain and for study of apoptosis with TUNEL stain (terminal deoxynucleotidyl-mediated deoxyuridine triphosphate nick end labeling stain) [18] using a commercially available kit (Detection Kit POD, Boehringer Mannhein, Mannhein, Germany). Apoptotic nuclei were identified by detected fluorescence.

### Human study

**Figure 2:**
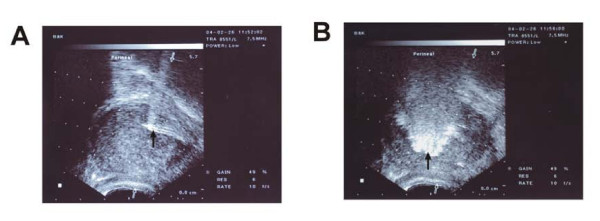
A. Confirmation of needle (black arrow) within the human prostate, longitudinal view. B. Diffusion of hyperechoic BTX-A (black arrow) over the prostate immediate post injection.

The effects on the human prostate and lower urinary tract symptoms were evaluated on 8 men with symptomatic BPH who had large prostate volume (mean 61.6 ml) and inadequate responses or intolerable side effects to α-blockers (Table 1). The study was approved by the institutional review board of Chang Gung Memorial Hospital Kaohsiung and informed consent was obtained. Eight men, mean age 71.1 ± 1.9 years, were treated. All patients had a prostate volume of greater than 40 ml and a peak urine flow rate less than 12 ml/s. All men had a benign digital rectal prostate exam and did not suspect clinical malignancy. Patient 3 had PSA value at 9 ng/ml but rejected biopsy. Patient 4 and 5 had been experiencing chronic urinary retention for more than 1 month and were treated with an indwelling Foley catheter. All patients received perioperative antibiotics with cefazolin 1 g (i.v.) and intravenous sedation with propofol 50 mg in the lithotomy position. Transperineal injection was done under TRUS guidance (Fig. 2) [17]. As previous study described, each lobe of the prostate received 100 U of BTX-A (Botox^®^, Allergan) dissolved in 4 ml of normal saline [13]. Two injections of equal volume (2 ml) were given in each lobe. The total dose of BTX-A was 200 U for each patient. Urethral catheter drainage was not performed postoperatively, except in two patients with chronic indwelling urethral catheters. These two men with urinary retention were able to urinate spontaneously 1 week post BTX-A injection. Patient 4 who was lack of pre-injection PSA data and was found with high PSA after removal of Foley catheter received biopsy and proved adenocarcinoma 3 months after injection. He received brachytherapy and was followed-up at our out patient clinic.

All men were evaluated pre and postoperatively for International Prostate Symptom Score (IPSS, 0–35, mild 0–7, moderate 8–18, severe ≥ 19), quality of life indices (QOL, 0–6, indicating increasing severity of symptoms and low quality of life) [19], peak urinary flow rates, post-void residual volume detected by ultrasound, and prostate volume by TRUS (0.52 × Length × Height × Width). Results were evaluated one month after the procedure. Quantitative data are expressed throughout this paper as means plus or minus standard error. Statistical analyses were performed using Student's t test for paired data with p < 0.05 considered significant.

## Results

### Animal study

**Figure 3:**
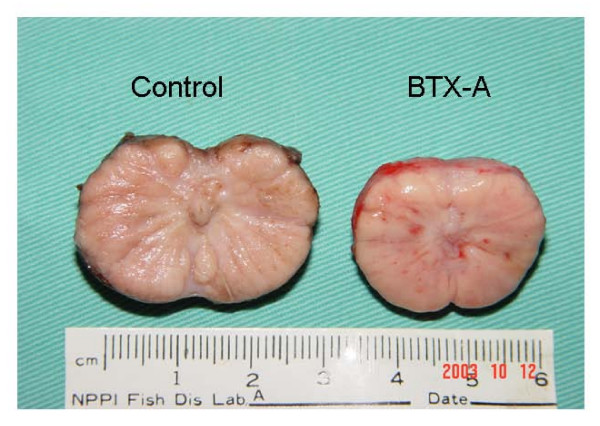
Coronal section of canine prostate one month after BTX-A or saline injection. Smaller in size and less indurations after BTX-A injection (B) than the control (A).

**Figure 4:**
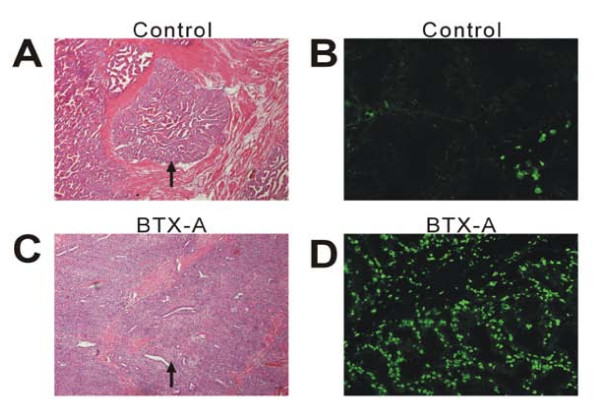
Photomicrographs and terminal deoxynucleotidyl-mediated deoxyuridine triphosphate nick end labeling (TUNEL) staining of prostate sections after saline (A,B) and BTX-A (C,D) injection at 1 month. Significant glandular proliferation with papillary infolding in the lumen was seen in the control canine (A). Atrophy change of glandular component with flattening of the lining epithelium was seen in the BTX-A treated canine (C). Very few apoptotic nuclei are recognizable in the control animal (B). Strong fluorescence of apoptotic nuclei was seen in the BTX-A treated animal at 1 month. A, C magnification × 40; B, D magnification × 100.

None of the dogs suffered any complications including infection, incontinence, urinary retention, stones, and change in ambulation or weight loss. The average prostate volume was not significantly changed after saline or BTX-A injection (from 11.8 ± 0.9 cc to 12.5 ± 1.1 cc and 11.0 ± 0.5 cc to 10.1 ± 0.4 cc, respectively). However, gross examination of the prostate revealed smaller in size and less indurated after BTX-A injection than the control (Fig. 3). The H&E stained sections of the prostate from the control animals of the 1 and 3 months groups revealed hypertrophied glands (Fig. 4A). However, the H&E stained sections of the BTX-A treated animals from the 1 and 3 month post BTX-A treated groups revealed a degeneration of the prostate glands with similar results in both groups (Fig 4C).

The TUNEL staining of sections of control animals (1 and 3 month groups) revealed only a small amount of apoptosis (Fig. 4B). The TUNEL stained sections of tissue of BTX-A treated animals revealed fluorescence in the nuclei throughout the evaluated tissue, but fluorescence was predominantly seen in the nuclei of the glands (Fig. 4D). This significant glandular apoptosis was seen at both 1 and 3 month after intraprostatic BTX-A injection.

The proportion of TUNEL positive nuclei per 500 nuclei was quantified at ×400 magnification using a 10 × 10 grid in the eyepiece in 4 fields. The mean apoptotic cells in the BTX-A treated group and control group were 187.5 ± 20.4 and 6.5 ± 2.1, respectively (p < 0.05).

### Human study

The follow-up period ranged from 3 to 8 months, mean 4.8 ± 0.6 months. No stress incontinence, retrograde ejaculation, erectile dysfunction, or systemic side effects were observed. Most patients reported improvement starting from 3 to 7 days post BTX-A treatment, reaching maximal effect in about 1 month, and maintaining effects as long as eight months.

At one month follow-up, the prostate volume, mean symptom score and quality of life index were significantly reduced by 18.8% (from 61.6 ± 8.7 to 50.0 ± 5.9 ml, p < 0.05), 73.1% (from 19.0 ± 1.8 to 5.1 ± 2.0, p < 0.05) and 61.5% (from 3.9 ± 0.3 to 1.5 ± 0.2, p < 0.05) respectively. The maximal flow rate was increased by 72.0% (from 7.5 ± 1.8 to 12.9 ± 0.5 ml/sec, p < 0.05). The residual urine was decreased by 86.2% (from 177.6 ± 71.7 to 24.5 ± 4.5 ml, p = 0.064). At three month follow-up, the effects were maintained and the prostate volume, mean symptom score, quality of life index, maximal flow rate, and residual urine were 49.5 ± 5.9 ml, 4.0 ± 0.9, 1.6 ± 0.3, 13.0 ± 0.5 ml/sec, and 22.1 ± 3.0 ml, respectively.

## Discussion

Although the etiology of BPH remains unknown, it has been suggested that BPH has two components: a static component that is related to prostatic enlargement and a dynamic component that reflects contraction of smooth muscle within the gland [1,12]. The static component is under parasympathetic control and regulated by androgen, while the stromal smooth muscle is sympathetically influenced [20,21]. Thus, drugs that relax smooth muscle within the prostate, such as α-adrenergic receptor blockers, and drugs that shrink the volume, such as 5 α reductase inhibitors, are used for the treatment of BPH.

Recent evidence suggests that BPH could be originated from neural dysregulation of the prostate and alterations in local neuropeptides [20]. The prostatic neuroendocrine peptides may regulate the synthesis of prostatic secretory product, growth, and function [20,21]. Thus, BTX-A which has a cholinergic receptor predilection in its action on nerve terminals as well as inhibition of release of noradrenalin might regulate the neural control of prostate and relieve the symptoms of benign prostatic obstruction.

There are some differences between the canine and human prostate, nevertheless, experimental findings on the canine prostate can be regarded as valuable information. Previous studies have used the canine mode to elucidate the effects of orchiectomy and androgen on the prostate [15,22]. In the canine model, we used BTX-A 100 U for prostate injection, which dose might be about 2 times of the dose used in human (100 U for 15 kg in canine/200 U for 60 kg in human). However, the component of human prostate is different from the canine prostate. Species differences might have different responses to BTX-A treatment [23], therefore, the selected dose of BTX-A 100 U for the canine model cannot be completely interpreted to the human dose. Our present results suggest that injection of BTX-A into the canine prostate induced marked atrophy and diffuse apoptosis of prostate glands. The effect persisted for at least 3 months without any notable side effects. Doggweiler suggested that denervation can alter growth-factor expression in the prostate and resulted in programmed cell death [11]. Kyprianou demonstrated that the suppression of sympathetic tone on the prostate induced prostate apoptosis [24]. Expression of specific prostate apoptosis related genes, such as bcl2 and transforming growth factor-β has been implicated in the pathogenesis of BPH [24]. Thus, apoptotic changes in the prostate after BTX-A treatment are likely to be related with reduced neurotrophic influence on the gland. Therefore, the induction of apoptosis may emerge as an attractive target for the management of BPH. As BPH might arise from neural dysregulation, BTX-A could potentially affect these neural mechanisms. Further study with staining of various neuropeptide markers in the dog prostate is undergoing for future report.

Although one of our patients did not reveal volume shrinkage, all of the 8 patients had improved in maximal flow rate, residual urine, IPSS score and quality of life indices, and had no side effects after BTX-A injection. The pathological features of BPH are heterogenous and include varying abnormalities of epithelium, smooth muscle and fibrous stroma. The relative proportion of epithelium and stroma is from 1:2 to 1:5 [24]. It is possible that the predominant component of the BPH nodule may determines the response to specific therapy, for example, smooth muscle predominant nodules would respond to α-blockers and epithelial nodules to androgen-deprivation therapy [1,24]. The possibility of BTX-A effects on the dynamic component of BPH might explain the patient without volume shrinkage, but improved in lower urinary symptoms. Taken together, BTX-A might reduce the benign prostatic obstruction no matter what the detrusor contractility is.

The effects of denervation provided by the BTX-A will wear off, as new axons re-sprout in approximately 6 months [7]. Phelan et al reported that BTX-A effects on sphincter injection have prolonged subjective clinical efficacy beyond 6 months [8]. The beneficial results of the present study were evident within 1 month, and they continued throughout the follow-up period. The duration of BTX-A effects might depend on the different characteristic of targeted tissue, therapeutic dose, and intervals. Patients will determine if and when subsequent injections are necessary. Since the application of BTX-A on the treatment of lower urinary dysfunction is currently off-level use in Taiwan, it was necessary to be approved by the institutional review board for the human use and animal study. In support of evidence based medicine practices, caution should be applied until larger randomized clinical studies are completed that will guide physicians in making decisions about the use of botulinum toxin in the prostate.

## Conclusion

In conclusion, our preliminary results suggest that BTX-A induces prostate atrophy and apoptosis in the canine and improves lower urinary tract symptoms in men with lower urinary tract symptoms due to benign prostatic hyperplasia. Intraprostate BTX-A injection may be a promising, reversible and alternative treatment for refractory BPH.

## Competing interests

The author(s) declare that they have no competing interests.

## Authors' contributions

YC & MB composed the projects and write paper; Y and PH help to composed the project; CC do the histopathological review; CH & HJ do the animal study

## Pre-publication history

The pre-publication history for this paper can be accessed here:



## Figures and Tables

**Table 1:** Patient profiles and results after botulinum toxin-A treatment.

Patient	Age (yr)	Prostate volume (ml) Pre/Post	PSA (ng/ml) Pre/Post	IPSS Pre/Post	QOL index Pre/Post	Peak Flow (ml/s) Pre/Post	Residual urine (ml) Pre/Post	Follow-up (month)
1	75	45.0/40.6	4.1/1.6	16/2	3/1	6/11	73/20	8
2	60	54.3/40.7	4.1/3.2	22/15	3/2	9.8/13	138/20	6
3	77	116.0/85.0	9.0/6.6	18/13	4/2	12/14	18/15	6
4	75	51.8/36.4	-/20.7	26/2	5/1	0/12	500/24	4
5	73	76.2/61.4	-/5.2; 4.4	25/5	5/2	0/12	500/40	4
6	70	46.3/39.3	3.6/-	11/1	3/1	12/15	72/17	4
7	72	61.8/54.9	3.3/3.5	19/2	4/2	11/14	100/48	3
8	67	41.3/41.0	2.0/2.4	15/1	4/1	9/12	20/12	3

IPSS- international prostate symptom score.

QOL- quality of life.

-: lack of data.
